# Low-Energy Extracorporeal Shock-Wave Therapy in the Treatment of Chronic Insertional Achilles Tendinopathy: A Case Series

**DOI:** 10.1155/2016/7123769

**Published:** 2016-10-24

**Authors:** Vito Pavone, Luca Cannavò, Antonio Di Stefano, Gianluca Testa, Luciano Costarella, Giuseppe Sessa

**Affiliations:** Dipartimento di Chirurgia Generale e Specialità Medico Chirurgiche, Sez. Ortopedia e Traumatologia A. O. U. Policlinico-Vittorio Emanuele, Catania, Italy

## Abstract

*Introduction*. We report the results of a series of 40 patients with chronic insertional Achilles tendinopathy treated with low-energy ESWT after the failure of a 3-month program of eccentric exercises alone.* Methods and Materials*. 40 patients, 28 (70%) males and 12 (30%) females, were treated between January and December 2014. All patients were previously treated with only eccentric exercises for a 3-month period. The treatment protocol included 4 sessions of ESWT with a 2-week interval, from 800 shots in each one (4 Hz, 14 KeV), together with eccentric exercises. Visual Analogue Scale (VAS) and American Orthopedic Foot and Ankle Society (AOFAS) Hindfoot score were recorded.* Results*. At the 12-month follow-up, 26 (65.0%) patients did not complain about pain (VAS < 2), 11 (27.5%) patients got back to normal activities despite residual pain (VAS 2–4), and 3 (7.5%) of the patients still complained about pain (VAS > 4). There was no significative improvement in both scores after eccentric exercises alone. Mean VAS improvement was 5.8 ± 1.3 SD points (*P* < 0.001). Mean AOFAS Hindfoot score improvement was 19.8 ± 5.0 SD points (*P* < 0.001).* Conclusions*. ESWT is recommended, in combination with an eccentric exercise program, in patients with chronic Achilles tendinopathy being both insertional and not.

## 1. Introduction

Chronic Achilles tendinopathy is one of the most frequent injuries during sport and physical practice representing 5%–18% of all running injuries [1]. This condition, characterized by chronic pain, has a great impact, especially in professional athletes [2]. Chronic pain and long recovery time have stimulated interest around the various treatment options. Among the various therapies offered, one with Extracorporeal Shock-Wave Therapy (ESWT) seems to be an effectiveness support to other noninvasive therapies [3] like eccentric exercises, although there is no consensus in literature around the ESWT application protocol (number of strokes, energy, and use of anesthetics). In this article, we report the results of a series of 40 patients suffering from resistant chronic insertional Achilles tendinopathy, treated with a low-energy ESWT protocol, after the failure of a 3-month program of eccentric exercises alone.

## 2. Methods and Materials

40 patients, 28 (70%) males and 12 (30%) females, were treated between 1 January and 31 December 2014 for chronic insertional Achilles tendinitis according to* International Classification of Diseases, 10th Revision* procedure codes (code M76.6) [4]. The study-enrolled patients showed persistent pain for more than 3 months in the insertional region of Achilles tendon, especially during walking and physical activities.

The study was approved by local ethical committee and it is in accordance with the Declaration of Helsinki. The enrolled patients came from Outpatient Department of the Orthopedics Clinic, University of Catania (Catania, Italy). All the treatments were performed by two of us (V.P and L.Co.), the data were abstracted by two of us (A.Ds and G.T.) and the outcome measurements and statistical analysis were undertaken by one of us (L.Ca.).

The mean age was 41.0 years (range 32–56). The right tendon was involved in 25 (62.5%) cases and the left in 15 (37.5%) cases (Table 1). Before starting ESWT treatment, an X-ray exam of the two feet in anteroposterior and lateral view was carried out.

Patients started our protocol after at least 3 months of therapy with Non-Steroidal Anti-inflammatory Drugs (NSAIDs) for 4–7 days and stretching and eccentric exercises for 50 min a day four times a week. Before treatment with ESWT, 23 (57.5%) patients were treated with therapy and 17 (42.5%) with diathermic treatment.

The overuse and the possibility of tendon-shoe friction were taken into consideration before starting treatment.

The treatment protocol included stretching and eccentric exercises for 50 min a day four times a week, use of ice before and after every session, and treatment with low-energy radial ESWT. ESWT was carried out in 4 sessions, with a 2-week interval. Electromedical device OssaTron®, at a frequency of 4 Hz and a voltage of 14 KeV with 800 shots each session, was used for the treatment.

Visual Analogue Scale (VAS) to measure pain and American Orthopedic Foot and Ankle Society (AOFAS) Hindfoot score, to evaluate alignment and functional outcome, were performed. VAS and AOFAS Hindfoot score were registered at the end of the eccentric exercises period, at the beginning of ESWT + eccentric exercises protocol, and then at 2, 6, and 12 months.

Patients with cardiac disease, with pacemaker, in treatment with anticoagulants, in treatment or with chronic history of neoplasm, diabetes, or rheumatologic diseases, in treatment with corticosteroids, or with fluoroquinolones in the last 2 years and patients with vascular diseases of the lower extremities were excluded from the study.

Two-tailed Student's *t*-test was performed to compare the difference between VAS and AOFAS Hindfoot score along the study period. For statistical analysis, Ministat was employed.

Limits of the study were the small sample size and the absence of control group together with the short-term follow-up.

## 3. Results

At the 12-month follow-up, 26 (65.0%) patients did not complain about pain (VAS < 2), 11 (27.5%) patients got back to normal daily activities and sports despite residual pain (VAS 2–4), and 3 (7.5%) of the patients still complained about pain (VAS > 4).

Mean VAS value at the beginning of eccentric exercises program was 7.7 ± 0.5 DS and after eccentric exercises was 7.4 ± 0.6 SD (*P* > 0.05). At the beginning of ESWT + eccentric exercises treatment, VAS score was 7.6 ± 0.6 DS, 3.8 ± 0.7 SD at 2 months, 2.8 ± 0.7 SD at 6 months, and 1.9 ± 1.2 SD after 12 months, with a mean improvement at the end of treatment of 5.8 ± 1.3 SD points. The results observed were statistically significant (*P* < 0.001).

Mean AOFAS score at the beginning of eccentric exercises program was 71.5 ± 5 SD and it was 71.0 ± 4.1 SD at the end (*P* > 0.05). Mean AOFAS Hindfoot score at the beginning of treatment was 71.4 ± 4.6 SD, 85.2 ± 4.1 SD at 2 months, 89.2 ± 3.6 SD at 6 months, and 91.3 ± 3.8 SD at 12 months, with a mean improvement of 19.8 ± 5.0 points (*P* < 0.001).

At X-ray examination, in 10 (25%) cases, calcification in the insertional region and, in 15 (37.5%) cases, the presence of Haglund's deformity were reported.

## 4. Discussion

Therapy with low-energy ESWT has been shown, in our series, to be effective in both reducing pain and function recovery after the failure of only eccentric exercises program and occasional NSAID treatment. The last 10 years' literature supports our conclusions (Table 2).

In 2005, Costa et al. published a randomized clinical trial in which they verified the use of ESWT administered 1 time per month for 3 months [9]. The study did not show a real beneficial effect on pain and the use of ESWT was not supported, probably because the time between the different sessions was too long. After this first trial, the trend in literature started to change. In fact, a randomized trial, published by Rasmussen et al. in 2008, revealed a beneficial effect of ESWT on functional recovery despite not significant results on pain [5]. The study did not differentiate between insertional and noninsertional Achilles tendinopathy. The protocol of treatment used was 2,000 strikes (0 : 12 to 0 : 51 mJ/mm^2^, 50 Hz) in 4 sessions once a week. They explain the poor effect on pain with the earlier recovery to a full level activity. Simultaneously, between 2006 and 2008, Furia published two case-control trials [7, 10]. The author evaluated the effectiveness of the treatment, respectively, on insertional and noninsertional Achilles tendinopathy, using a single dose of high-energy ESWT: 3000 shots (0.21 mJ/mm and 604 mJ/mm). These studies showed a beneficial effect on pain and supported the use of ESWT in both insertional and nooinsertional Achilles tendinopathy. Then, in 2007, Rompe et al. published a randomized case-control trial comparing eccentric exercises, low-energy ESWT, and “wait and see” in treatment of chronic tendinopathy of Achilles tendon body. The ESWT protocol was delivered in 3 sessions of 2000 weekly interval shots (0.1 mJ/mm^2^; 8 Hz) [11]. Improvement in pain symptoms in the group with eccentric exercises and in the group treated with ESWT in comparison with simple observation was found. In 2008, Rompe et al. again in treating chronic Achilles insertional tendinopathy released another randomized case-control trial comparing the effect of eccentric exercise versus low-energy radial ESWT [6]. Three sessions of 2000 strikes once a week (0.1 mJ/mm^2^; 8 Hz) were administered. Excellent results in terms of both pain and recovery were observed in both groups, with significantly better results in ESWT group compared with only eccentric exercises. Recently, a very interesting prospective study was published by Taylor et al. in 2016, who evaluate the effectiveness of ESWT in the treatment of refractory Achilles tendinopathy with a 24-month follow-up [8]. They make a distinction between noninsertional Achilles tendinopathy (NAT) and insertional Achilles tendinopathy (IAT) and analyze the results among different age groups. They found pain and functional improvement across all age groups. Unfortunately no control group was compared in this study.

If we summarize the results of these various studies, it is worth mentioning two meta-analyses. The first, by Al-Abbad and Simon in 2013, evaluated the effectiveness of ESWT in treating chronic tendinopathy Achilles [1]. Six studies are examined. With the selection of 6 studies, the authors concluded that the ESWT was effective with a level of evidence 1, particularly when associated with an eccentric exercise program, in the treatment of chronic Achilles tendinopathy. The second meta-analysis, published by Gerdesmeyer et al. in 2015, examined 6 clinical trials [3]. Also in this study authors conclude that ESWT is effective, in terms of pain and functional recovery, in the treatment of Achilles chronic tendinopathy of both insertional and noninsertional condition. No evidence of a specific recommended protocol was reported.

## 5. Conclusions

The ESWT is recommended, especially in combination with an eccentric exercise program and in association with other not invasive treatments of chronic Achilles tendinopathy that are both insertional and not. Further studies are needed requirements to establish the most effective protocol.

## Figures and Tables

**Table 1:** Study group.

Patients	40
Feet	40
Males	28 (70%)
Females	12 (30%)
Mean age	41.0 (range 32–56)
Right	25 (62.5%)
Left	15 (37.5%)
Laser therapy before ESWT treatment	23 (57.5%)
Diathermic therapy before ESWT treatment	17 (42.5%)

**Table 2:** Case series, VAS score during activity.

Authors	N. cases	Follow-up (months)	Treatment	ESWT parameters	Pain improvement	Functional improvement
Rasmussen et al. 2008 [5]	48 (24 + 24)	3	ESWT versus placebo	Low-energy radial ESWT, 4 sessions, 2,000 shots, 0.12–0.51 mJ/mm^2^, 50 Hz	/	AOFAS +18 points (*P* < 0.05)
Rompe et al. 2008 [6]	50 (25 + 25)	12	ESWT versus eccentric exercises	Low-energy ESWT, 3 sessions, 2000 shots, 0.12 mJ/mm^2^	NRS −3.0 ± 2.3 (0–8)	VISA-A +27 points (*P* < 0.001)
Furia 2006 [7]	68 (35 + 33)	12	ESWT versus control	High-energy ESWT, 1 dose, 3000 shocks, 0.21 mJ/mm^2^, total energy flux density, 604 mJ/mm^2^	VAS 7.0 (control) and 2.8 (ESWT) (*P* < 0.001)	Roles-Maudsley (*P* < 0.001)
Taylor et al. 2016 [8]	56 (46)	24	ESWT	Low-energy radial ESWT, 3 sessions, 2000 shots, 10 Hz, 1.5–2.5 bar	VAS −4.8 (NAT) and −3.9 (IAT) (*P* < 0.01)	VISA-A +66 points (*P* < 0.001)
The present study	40	12	ESWT + eccentric exercises	Low-energy radial ESWT, 4 sessions, 800 shots, 4 Hz, 14 KeV	VAS −5.8 ± 1.2 SD (*P* < 0.001)	AOFAS +19.8 ±5.0 SD (*P* < 0.001)
